# Supplementary material for: Meta-analyses and Forest plots using a microsoft excel spreadsheet: step-by-step guide focusing on descriptive data analysis
Source: BMC Res Notes. 2012 Jan 20;5:52. doi: 10.1186/1756-0500-5-52 (PMC3296675; doi:10.1186/1756-0500-5-52)
Supplement: Additional file 2 — CMA calculations fixed effect. This is a portable document format (pdf) of the calculations performed by the software Comprehensive Meta-Analysis, when calculating the effect summary using fixed effect model. It is provided so readers may compare the calculations and results obtained using Microsoft Excel spreadsheet and the commercial software. [file 1756-0500-5-52-S2.PDF]

Model	Study name	Calculations (Fixed)													
		Point	Study Variance	Tau^2 Within	Tau^2 Between	Total Variance	IV-Weight	W	T*W	T^2*W	W^2	C	Q	Q df	W^3
	1,00000000	0,20179820	0,00010079	0,00000000	0,00000000	0,00010079	9920,80206	9920,80206	2002,00000	403,999996	98422313,6	89021,9012	17,7311819	9,00000000	976428293
	2,00000000	0,16430590	0,00046545	0,00000000	0,00000000	0,00046545	2148,43167	2148,43167	353,000000	57,9999827	4615758,66	89021,9012	17,7311819	9,00000000	991664211
	3,00000000	0,19809320	0,00020984	0,00000000	0,00000000	0,00020984	4765,43364	4765,43364	944,000000	186,999980	22709357,8	89021,9012	17,7311819	9,00000000	108219937
	4,00000000	0,18297870	0,00012977	0,00000000	0,00000000	0,00012977	7705,81493	7705,81493	1410,00000	257,999967	59379583,8	89021,9012	17,7311819	9,00000000	457568084
	5,00000000	0,21445550	0,00010197	0,00000000	0,00000000	0,00010197	9806,23019	9806,23019	2103,00000	450,999916	96162150,6	89021,9012	17,7311819	9,00000000	942988185
	6,00000000	0,17768600	0,00007342	0,00000000	0,00000000	0,00007342	13619,5310	13619,5310	2420,00000	430,000120	185491626,	89021,9012	17,7311819	9,00000000	252630897
	7,00000000	0,21580650	0,00006961	0,00000000	0,00000000	0,00006961	14364,7202	14364,7202	3100,00000	669,000150	206345187,	89021,9012	17,7311819	9,00000000	296409089
	8,00000000	0,20717130	0,00082538	0,00000000	0,00000000	0,00082538	1211,55777	1211,55777	251,000000	51,9999963	1467872,25	89021,9012	17,7311819	9,00000000	177841204
	9,00000000	0,19704750	0,00002529	0,00000000	0,00000000	0,00002529	39533,6149	39533,6149	7790,00000	1535,00002	156290671	89021,9012	17,7311819	9,00000000	617873523
	10,0000000	0,18916800	0,00014848	0,00000000	0,00000000	0,00014848	6734,75429	6734,75429	1274,00000	241,000032	45356915,3	89021,9012	17,7311819	9,00000000	305467680
		1,94851080	0,00215004	0,00000000	0,00000000	0,00215004	109810,890	109810,890	21647,0000	4285,00016	228285748	89021,9012	17,7311819	9,00000000	700801194

Model	Study name	Calculations (Fixed)						
		I^2	B	K	Summary Point	Summary Variance	Group T^2	Group T^2 Variance
	1,00000000	49,2419623	40,3015609	10,0000000	0,19712980	0,00000910	0,00009807	0,00000001
	2,00000000	49,2419623	40,3015609	10,0000000	0,19712980	0,00000910	0,00009807	0,00000001
	3,00000000	49,2419623	40,3015609	10,0000000	0,19712980	0,00000910	0,00009807	0,00000001
	4,00000000	49,2419623	40,3015609	10,0000000	0,19712980	0,00000910	0,00009807	0,00000001
	5,00000000	49,2419623	40,3015609	10,0000000	0,19712980	0,00000910	0,00009807	0,00000001
	6,00000000	49,2419623	40,3015609	10,0000000	0,19712980	0,00000910	0,00009807	0,00000001
	7,00000000	49,2419623	40,3015609	10,0000000	0,19712980	0,00000910	0,00009807	0,00000001
	8,00000000	49,2419623	40,3015609	10,0000000	0,19712980	0,00000910	0,00009807	0,00000001
	9,00000000	49,2419623	40,3015609	10,0000000	0,19712980	0,00000910	0,00009807	0,00000001
	10,0000000	49,2419623	40,3015609	10,0000000	0,19712980	0,00000910	0,00009807	0,00000001
		49,2419623	40,3015609	10,0000000	0,19712980	0,00000910	0,00009807	0,00000001
