# Supplementary material for: Meta-analyses and Forest plots using a microsoft excel spreadsheet: step-by-step guide focusing on descriptive data analysis
Source: BMC Res Notes. 2012 Jan 20;5:52. doi: 10.1186/1756-0500-5-52 (PMC3296675; doi:10.1186/1756-0500-5-52)
Supplement: Additional file 3 — CMA calculations random effects. This is a portable document format (pdf) of the calculations performed by the software Comprehensive Meta-Analysis, when calculating the effect summary using random effects model. It is provided so readers may compare the calculations and results obtained using Microsoft Excel spreadsheet and the commercial software. [file 1756-0500-5-52-S3.PDF]

Model	Study name	Calculations (Random)													
		Point	Study Variance	Tau^2 Within	Tau^2 Between	Total Variance	IV-Weight	W	T*W	T^2*W	W^2	W^3	C	Q	Q df
	1,00000000	0,20179820	0,00010079	0,00009807	0,00000000	0,00019887	5028,22508	5028,22508	1014,68677	204,761964	25283047,5	127128853	39167,2695	9,03274464	9,00000000
	2,00000000	0,16430590	0,00046545	0,00009807	0,00000000	0,00056353	1774,51317	1774,51317	291,562985	47,9055186	3148897,02	558775927	39167,2695	9,03274464	9,00000000
	3,00000000	0,19809320	0,00020984	0,00009807	0,00000000	0,00030792	3247,55961	3247,55961	643,319476	127,437213	10546643,4	342508533	39167,2695	9,03274464	9,00000000
	4,00000000	0,18297870	0,00012977	0,00009807	0,00000000	0,00022785	4388,82997	4388,82997	803,062403	146,943314	19261828,5	845368904	39167,2695	9,03274464	9,00000000
	5,00000000	0,21445550	0,00010197	0,00009807	0,00000000	0,00020005	4998,62490	4998,62490	1071,98260	229,892565	24986250,9	124896896	39167,2695	9,03274464	9,00000000
	6,00000000	0,17768600	0,00007342	0,00009807	0,00000000	0,00017150	5830,80182	5830,80182	1036,05185	184,091909	33998249,8	198237057	39167,2695	9,03274464	9,00000000
	7,00000000	0,21580650	0,00006961	0,00009807	0,00000000	0,00016769	5963,24142	5963,24142	1286,90626	277,722736	35560248,3	212054345	39167,2695	9,03274464	9,00000000
	8,00000000	0,20717130	0,00082538	0,00009807	0,00000000	0,00092346	1082,88077	1082,88077	224,341817	46,4771859	1172630,76	126981931	39167,2695	9,03274464	9,00000000
	9,00000000	0,19704750	0,00002529	0,00009807	0,00000000	0,00012337	8105,43802	8105,43802	1597,15629	314,715655	65698125,6	532512085	39167,2695	9,03274464	9,00000000
	10,0000000	0,18916800	0,00014848	0,00009807	0,00000000	0,00024656	4055,76599	4055,76599	767,221141	145,133688	16449237,7	667142592	39167,2695	9,03274464	9,00000000
		1,94851080	0,00215004	0,00098079	0,00000000	0,00313083	44475,8808	44475,8808	8736,29161	1725,08175	236105159,	138718882	39167,2695	9,03274464	9,00000000

Model	Study name	Calculations (Random)						
		I^2	B	K	Summary Point	Summary Variance	Group T^2	Group T^2 Variance
	1,00000000	0,36251049	9,06563041	10,0000000	0,19642762	0,00002248	0,00009807	0,00000001
	2,00000000	0,36251049	9,06563041	10,0000000	0,19642762	0,00002248	0,00009807	0,00000001
	3,00000000	0,36251049	9,06563041	10,0000000	0,19642762	0,00002248	0,00009807	0,00000001
	4,00000000	0,36251049	9,06563041	10,0000000	0,19642762	0,00002248	0,00009807	0,00000001
	5,00000000	0,36251049	9,06563041	10,0000000	0,19642762	0,00002248	0,00009807	0,00000001
	6,00000000	0,36251049	9,06563041	10,0000000	0,19642762	0,00002248	0,00009807	0,00000001
	7,00000000	0,36251049	9,06563041	10,0000000	0,19642762	0,00002248	0,00009807	0,00000001
	8,00000000	0,36251049	9,06563041	10,0000000	0,19642762	0,00002248	0,00009807	0,00000001
	9,00000000	0,36251049	9,06563041	10,0000000	0,19642762	0,00002248	0,00009807	0,00000001
	10,0000000	0,36251049	9,06563041	10,0000000	0,19642762	0,00002248	0,00009807	0,00000001
		0,36251049	9,06563041	10,0000000	0,19642762	0,00002248	0,00009807	0,00000001
